# Impact of short school closures (1–5 days) on overall academic performance of schools in California

**DOI:** 10.1038/s41598-022-06050-9

**Published:** 2022-02-08

**Authors:** Rebecca K. Miller, Iris Hui

**Affiliations:** 1 Huntington-USC Institute on California and the West, University of Southern California, 3502 Trousdale Parkway, Los Angeles, CA 90089 USA; 2 Bill Lane Center for the American West, Stanford University, 473 Via Ortega, Stanford, CA 94305 USA; 3 Emmett Interdisciplinary Program in Environment and Resources, Stanford University, 473 Via Ortega, Stanford, CA 94305 USA

**Keywords:** Environmental impact, Psychology and behaviour

## Abstract

Climate change impacts such as disasters and higher temperatures can disrupt academic learning and reduce academic performance. Here, we use two-ways linear fixed effects regression to estimate the effects of short-term school closures (1–5 days) due to wildfires, natural hazard impacts, infrastructure, and student safety on academic performance in California, focusing on mathematics and English scores from state assessments and college preparatory exams. Wildfires are responsible for the majority of school closures. Wildfires generate significant negative impacts on academic performance among younger students. We primarily find insignificant impacts on academic achievement due to school closures from other causes, including from the interaction between number of closure days and socioeconomic and racial/ethnic makeup of the school, across all causes. The effects of school closures lasting more than one week (6–10 days) are also generally insignificant, except for the negative impacts of wildfire closures on elementary school students. These results suggest that older students are resilient to most unexpected short-term school closures (1–5 days) or that teachers can make up lessons effectively after schoolwide closures.

## Introduction

Weather-related disasters are estimated to affect up to 175 million children globally each year, with the greatest impacts among poor children and those in developing countries^1^. Bad weather and disasters are responsible for the vast majority (93%) of unplanned school closures in the United States each year, followed by infrastructure concerns and illnesses^2^. Disasters can destroy or damage school infrastructure, thus preventing students from attending school, in addition to forcing migration or displacement from school districts^3,4^. Disasters can also threaten children’s perceptions of safety, security, and normalcy, resulting in higher rates of post-traumatic stress disorder, depression, moodiness, and other negative emotional reactions^4,5^. A rapid and safe return to school after a traumatic event like a wildfire is crucial to maintaining a stable support system for children, particularly because schools offer important routines and resources like lunches and counselors^6,7^. The rise of school violence such as school shootings or bomb threats^8^ may also result in reduced school performance and short-term trauma or PTSD^9^.

Prior studies have found strong associations between individual students missing school and lower performance on mathematics and reading assessments, even as early as pre-kindergarten^10–12^. Studies on absenteeism and academic performance tend to follow small cohorts of children over time to track the long-term impacts of missing school on an individual basis^13^. Absenteeism is most significantly associated with lower test scores among boys, low-income students, and minorities^14–18^. Missing school is also positively associated with a higher likelihood of dropping out of high school, which is further linked to higher rates of unemployment, lower median lifetime annual earnings, and greater likelihood of criminal activity and incarceration^19–21^.

By contrast, research on the effects of schoolwide closures on academic achievement is more limited and offers more divergent results. Prior literature on schoolwide closure days has focused on closures caused by disease outbreaks, extreme weather or disasters, school safety concerns, or other causes, with inconsistent findings (Supplementary Table 1). Most research on the educational impacts of the COVID-19 pandemic stem from model projections that predict significant learning losses, learning delays, and economic losses associated with missed school^22,23^. Studies on non-disease-related closures often use difference-in-differences and fixed effects to examine the impacts on specific educational outcomes like standardized test scores. Results from non-disease-related closure studies are inconsistent, with some studies identifying minimal or insignificant impacts on academic performance^24^, while others find significant, negative impacts from closures, particularly among low-income, minority, and younger student populations^25^ (Supplementary Table 1b).

Separate literature on standardized testing reveals that higher year-round and test-day temperatures both negatively affect education and academic performance, particularly among low-income and minority students^26–28^. Heat stress can produce significant negative impacts on complex cognitive performance^29^; minority and socioeconomically vulnerable populations are more likely to experience elevated heat exposure^30–32^. Therefore, both local weather conditions and schoolwide closures may influence student performance.

Here, we consider how disruptions from four causes of school closures may impact schoolwide academic performance based on two-ways linear fixed effects regression to control for school and year fixed effects. Drawing on the CalMatters Disaster Days dataset, we examine the effect of closures caused by wildfires, natural hazard impacts (referred to as “natural disasters and weather” in the Disaster Days dataset), infrastructure, and school safety on standardized test scores for statewide tests in English and mathematics and college preparatory exams (SAT, ACT, and AP exams). Here, natural hazard impacts include inclement weather, earthquakes, tsunami warnings, storms, and power outages associated with weather. We also consider the intersection of socioeconomic status and minority enrollment with the number of disruptions; prior research on standardized tests indicates disproportionate negative effects of both higher temperatures^26–28^ and some school closures^25^ on low-income and minority populations (Supplementary Table 1b). Our statistical analysis incorporates data on test scores from primary and secondary schools, student demographics and socioeconomic information, and school closures between the 2002–2003 and 2018–2019 academic years, based on available data. We use participation in free or reduced-price meal (FRPM) programs as a proxy for the percentage of low-income students at the school^33^. Throughout this paper, we use racial/ethnic designations as collected by the California Department of Education^34^. We use “disruption” and “school closure” interchangeably to refer to a school closure day.

Disruptions caused by wildfires, natural hazard impacts, infrastructure, and school safety may cause other negative impacts beyond school closures. For example, wildfires affect communities both directly due to immediate fire threat and danger and indirectly from widespread smoke impacts, possibly disrupting daily activities for prolonged periods of time and causing displacement, trauma, smoke inhalation, and home or economic disruptions^35^. Similarly, though most students are resilient to safety concerns like school shootings, others may express severe trauma-related symptoms^9^. We cannot isolate these additional impacts within our study, though they may introduce compounding or influencing effects on the relationship between school closures and academic achievement. Data limitations also prevent us from following cohorts of individual children over time and generating conclusive findings beyond the academic year of the closure, though closures may produce a lagged effect^36^.

This study explores two research questions. First, how frequently do disruptions result in schoolwide closures across public schools in California? Second, how do schoolwide closures due to different causes of disruption affect academic performance across public schools and within specific school populations at the school level? We also consider the impacts of school closures caused by quarantines and outbreaks to place our findings in the context of the COVID-19 pandemic. We examine school closure and academic performance data from public schools across seventeen academic years (2002–2003 through 2018–2019). Understanding whether short-term school closures impact academic performance at the schoolwide level by individual grades may provide valuable insights into how local and state education policies may need to adjust to support students and mitigate any educational impacts of school disruptions in the future.

## Results

### Frequency of school closures

**Figure 1:**
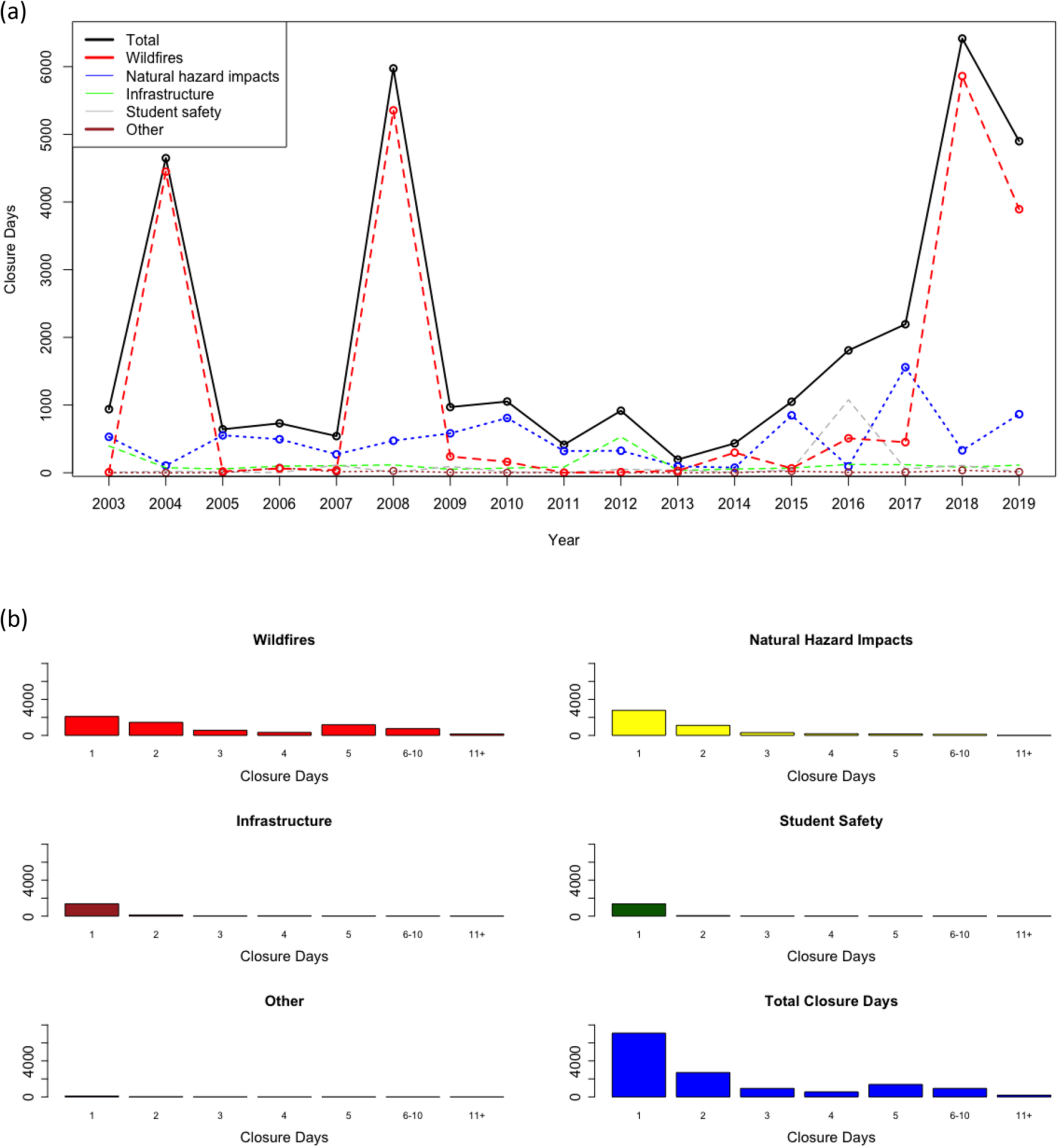
School closure days by cause. (**a**) Total school closure days across academic years ending in 2003–2019. (**b**) Histograms of frequency of school closure days by length of closures.

In the seventeen-year period between the 2002–2003 and 2018–2019 academic years, schools in California closed for a total of 33,819 days across 6664 individual schools (Table 1, Fig. 1a). Based on public school enrollment data from the California Department of Education, our dataset consisted of 106,168,312 students attending 15,738 individual schools over seventeen years. Closure days occurred as a result of wildfires (21,442 days; 63.4%), natural hazard impacts (8333; 24.6%), infrastructure (2171; 6.4%), student safety (1660; 4.9%), and other reasons such as memorial services or teachers’ strikes (213, 0.6%). Wildfire closures encompass both direct threats of a wildfire and smoke impacts or poor air quality. However, school closures remain rare. Schools in California in our dataset close for an average of 0.17 total days per year (range: 0.02 days, 2013; 0.55 days, 2018). On average, 813.8 (5.1%) public schools experience schoolwide closures per year (range: 142 schools in 2012–2013; 2262 schools in 2018–2019). Among schools that experienced schoolwide closures, closures lasted an average of 2.44 days, though wildfire-caused school closures lasted an average of 3.28 days (Fig. 1b). Wildfires were responsible for the vast majority of closures during the four academic years with more than 2500 total closure days (range: 3893 days in 2018–2019, 79.5%; 5860 days in 2017–2018, 91.3%).

**Table 1:** School closure data by cause between the 2002–2003 and 2018–2019 academic years.

Cause of school closure	Total days closed	Average closure length (days)	Fall semester days closed	Spring semester days closed	Student-days missed
Wildfires	21,442 (62.7%)	3.28	20,861 (78.7%)	581 (7.6%)	13,491,539 (72.3%)
Natural hazard impacts	8370 (24.5%)	1.78	2718 (10.3%)	5652 (73.7%)	3,208,048 (17.2%)
Infrastructure	2292 (6.7%)	1.33	1435 (5.4%)	857 (11.2%)	872,549 (4.7%)
Student safety	1660 (4.9%)	1.14	1292 (4.9%)	368 (4.8%)	1,002,170 (5.4%)
Other	419 (1.2%)	1.62	203 (0.8%)	216 (2.8%)	79,454 (0.4%)
Total	34,183	2.44	26,509	7674	18,653,777

The vast majority of schools (70.9%) close for no more than 2 days, with only 1136 (8.2%) schools closing for more than 5 days. Wildfires caused 80.5% (915) of the closures lasting more than 5 days and 86.1% of the school closures (162 of 188 closures) lasting more than 10 days. In addition, 12,699 (91.8%) of total closures are short-term and last up to 1 week (1–5 days), 948 (6.9%) are medium-term and last between 1 and 2 weeks (6–10 days), and 188 (1.4%) are long-term and last more than 2 weeks (11 + days).

Based on data collected by CalMatters, 78.0% of school closure days (26,384 days) occurred in the fall semester and 22.0% (7435 days) occurred in the spring semester (Table 1). Wildfires were the primary cause of school closure days during the fall semester, accounting for 20,861 (79.1%) fall closure days. Natural hazard impacts were the primary cause of school closure days during the spring semester, accounting for 5615 (75.5%) spring closure days.

Wildfires are also the biggest cause of missed student-days, referring to the cumulative number of school closures multiplied by the number of students enrolled at the affected schools. Between the 2002–2003 and 2018–2019 academic years, school closures resulted in 18,623,056 cumulative missed student-days, of which wildfires were responsible for 13,491,539 (72.4%) student-days (Table 1).

Schools with higher concentrations of white students have more school closures than any other subpopulation considered in our study, particularly from wildfires and natural hazard impacts (Table 2). Schools with higher concentrations of students receiving FRPMs and Hispanic or Latino, African-American, or Asian students have fewer school closures from wildfires and natural hazard impacts than those with lower concentrations. Infrastructure, student safety, and other causes are associated with the lowest number of school closures.

**Table 2:** Average number of closure days by closure cause by subpopulation quartile.

Closure cause	Quartile	FRPM	Hispanic	Black	Asian	White
Wildfires	1st	0.115	0.139	0.130	0.135	0.094
	2nd	0.135	0.150	0.081	0.059	0.158
	3rd	0.130	0.124	0.029	0.017	0.177
	4th	0.089	0.089	0.027	0.023	0.126
Natural hazard impacts	1st	0.034	0.079	0.038	0.038	0.022
	2nd	0.067	0.046	0.028	0.013	0.044
	3rd	0.068	0.034	0.020	0.029	0.077
	4th	0.037	0.015	0.004	0.014	0.152
Infrastructure	1st	0.010	0.021	0.010	0.009	0.007
	2nd	0.012	0.010	0.005	0.005	0.010
	3rd	0.012	0.006	0.001	0.003	0.018
	4th	0.011	0.006	0.002	0.000	0.041
Student safety	1st	0.005	0.007	0.009	0.009	0.012
	2nd	0.005	0.007	0.014	0.004	0.006
	3rd	0.009	0.008	0.021	0.003	0.007
	4th	0.018	0.017	0.019	0.003	0.006
Other	1st	0.001	0.002	0.001	0.001	0.001
	2nd	0.002	0.001	0.001	0.000	0.001
	3rd	0.001	0.001	0.000	0.000	0.002
	4th	0.001	0.000	0.000	0.000	0.006
Total	1st	0.164	0.248	0.188	0.193	0.136
	2nd	0.221	0.215	0.130	0.081	0.218
	3rd	0.220	0.173	0.072	0.052	0.281
	4th	0.157	0.128	0.050	0.040	0.331

Average closure days are rounded to the nearest thousandth.

### Impacts of school closures on academic performance

#### School disruptions by cause

We first tested whether school disruptions have an impact on academic performance. We consider the types of school disruption and an array of school-level characteristics (equation 1, see “Methods”). The dependent variables are the percentage of students who failed to meet the statewide English and Mathematics standards through the California Assessment of Student Performance and Progress (CAASPP) and scores on three college preparatory tests. We compute separate models for each grade, from grade 3 to grade 11 as well as all grades tested within a school (grade 13).

**Figure 2:**
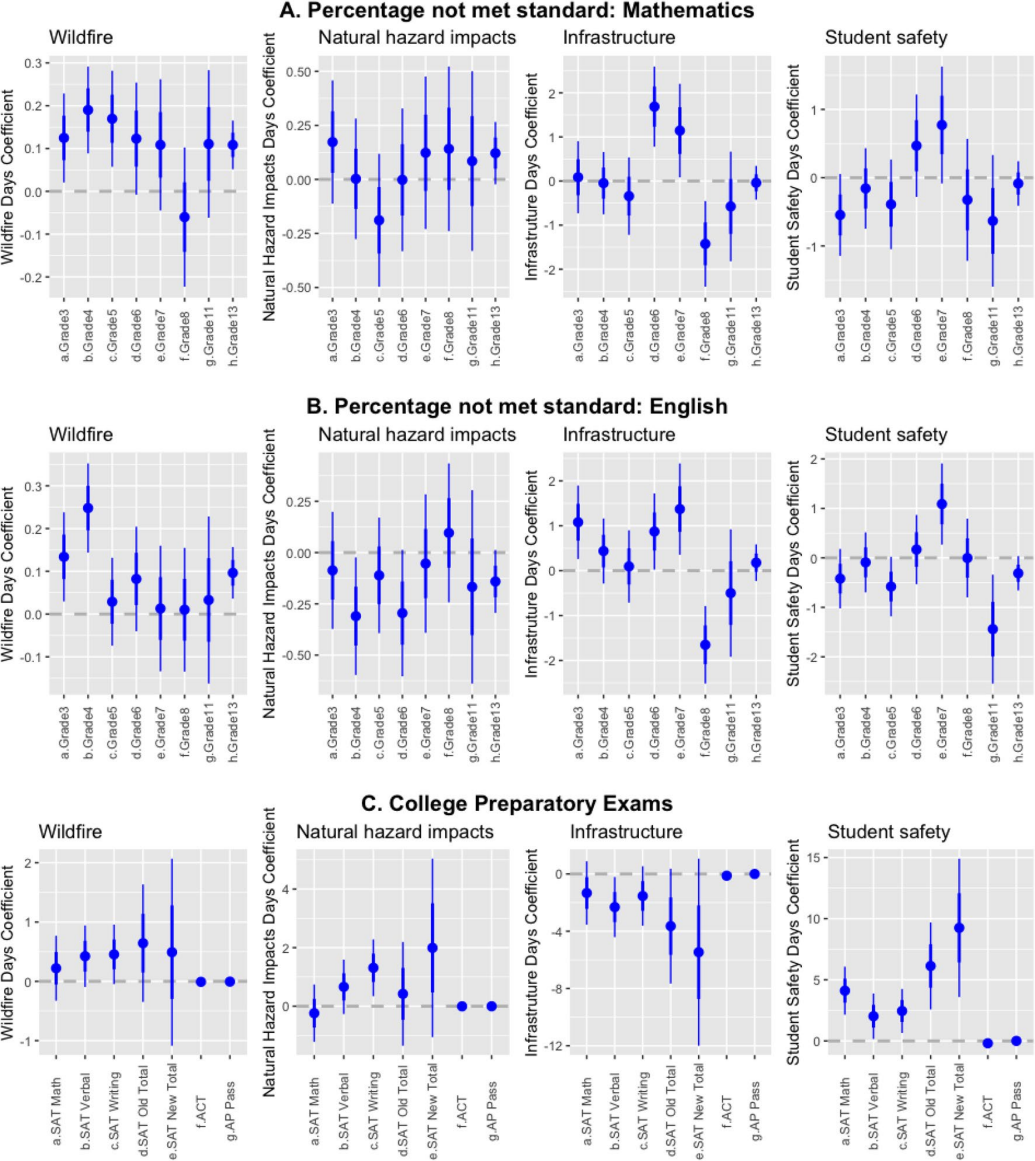
Effect of school closures on standardized test scores by closure cause for (**A**) grades 3–11 and all grades (grade 13) for mathematics, (**B**) grades 3–11 and all grades (grade 13) for English, and (**C**) college standardized exams. Lines indicate confidence intervals two standard deviations from the mean. Note that the y-axis scale varies among plots.

We find that the coefficients related to the CAASPP exams were statistically significant at the 0.05 level following wildfires, particularly for younger students in elementary school: worse 3rd, 4th, 5th, and 13th grade mathematics scores and worse 3rd, 4th, and 13th grade English scores. AP scores also declined following wildfire closures (Fig. 2). By contrast, natural hazard impacts closures are associated with only two statistically significant impacts: improved 4th grade English and improved SAT writing scores. Infrastructure closures primarily affect middle school students: worse 3rd grade English, worse 6th and 7th grade mathematics and English, improved 8th grade mathematics and English, and worse SAT English scores. Student safety closures are associated with worse 7th grade English, improved 11th grade English, and improved SAT mathematics, English, writing, and overall scores. Similar to wildfire closures, total closures primarily affect younger students, with worse scores in mathematics (3rd, 4th, 5th, 6th, 7th, and 13th grades), worse scores in English (3rd, 4th, and 13th grades), improved SAT English and writing, and worse AP exam scores.

#### Socioeconomic status

**Figure 3:**
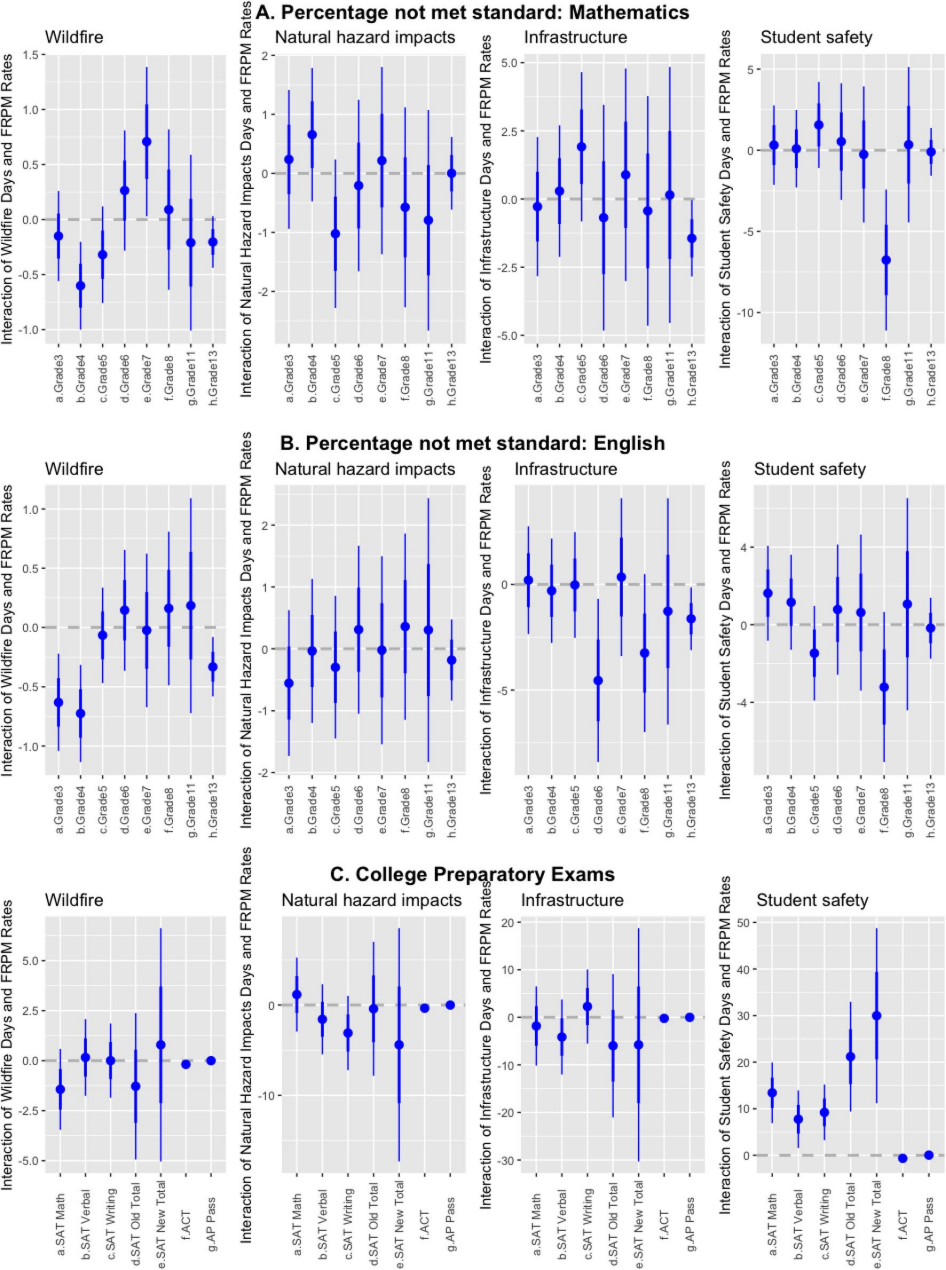
Effect of school closures caused by the interaction of closures and FRPM rates by closure cause on standardized test scores for (**a**) grades 3–11 and all grades (grade 13) for mathematics, (**b**) grades 3–11 and all grades (grade 13) for English, and (**c**) college standardized exams. Lines indicate confidence intervals two standard deviations from the mean. Note that the y-axis scale varies among plots.

The impacts of absenteeism and high temperatures disproportionately affect academic performance among low-income and minority students^10,13,26^. Therefore, we expect any negative impacts of school closures to concentrate among schools with higher proportions of low-income students and racial/ethnic minorities. We modify our model specification to consider schools with higher concentrations of students receiving free and reduced-price meals (FRPMs) as a proxy for socioeconomic status (equation 2, see “Methods”). The coefficients related to the CAASPP or college preparatory exams are statistically significant in six cases following wildfire closures: improved 4th grade mathematics; worse 7th grade mathematics; improved 3rd, 4th, and 13th grade English; and worse ACT scores (Fig. 3). Total closures are associated with the same impacts as wildfires in addition to improved 13th grade mathematics scores. We find no statistically significant impacts from natural hazard impacts on either CAASPP or college preparatory exam scores. In addition, we find minimal impacts from infrastructure (improved 6th and 13th grade English, improved 13th grade mathematics scores) and student safety closures (improved 8th grade mathematics, and improved SAT math, English, writing, overall, and AP exam scores). Overall, we find few significant impacts on schools with high populations of low-income students across the majority of statewide tests after closures from any cause or from total closure days.

#### Race and ethnicity

Next, we explore the impact of disruptions on schools with higher proportions of Hispanic or Latino, African-American, Asian, and white populations (equation 3, see “Methods”). We generally find significant impacts among younger students following wildfire and natural hazard impacts closures with minimal significant impacts from infrastructure or student safety disruptions.

Schools with higher concentrations of Hispanic or Latino students have improved 3rd and 4th grade English, improved 4th grade mathematics, worse 7th grade mathematics, and worse ACT scores following both wildfire and total closures (Supplementary Figs. 1, 5). Following natural disaster closures, we find improved 8th and 13th grade mathematics; improved 3rd grade English scores; and worse SAT English, SAT writing, and ACT scores. We find minimal impacts from infrastructure (improved 6th grade English, worse 13th grade English and mathematics scores) or student safety disruptions (improved 11th grade English, improved 13th grade mathematics and English, improved SAT scores). We find no other significant impacts among schools with higher concentrations of Hispanic or Latino students.

Among schools with higher concentrations of African-American students, we primarily find significant impacts following wildfires and natural hazard impacts disruptions, especially among younger students. Following wildfire closures, we find worse 3rd, 4th, 5th, 6th, and 13th grade mathematics scores and worse 3rd, 4th, 6th, and 13th grade English scores (Supplementary Fig. 2). Following natural disaster closures, we find improved 4th and 13th grade mathematics scores and improved 3rd, 4th, 5th, 7th, 8th, and 13th English scores. By contrast, we find minimal impacts from infrastructure closures (improved 4th grade English, worse 6th grade English scores), student safety closures (improved 6th and 8th grade mathematics scores), and total closures (improved 8th grade mathematics, worse 6th grade English scores).

Similarly, we primarily find significant impacts following wildfire and natural disaster closures among elementary school students at schools with higher concentrations of Asian students. Wildfire closures are associated with worse mathematics scores among 4th and 5th graders; worse English scores among 3rd, 4th, and 6th graders; and improved ACT scores (Supplementary Fig. 3). Following natural disaster closures, we find improved mathematics scores among 3rd, 4th, 5th, 6th, and 13th graders; improved English scores among 3rd, 4th, and 5th graders; and worse English scores among 8th graders. By contrast, we find few impacts from other causes: improved SAT writing (infrastructure), worse 8th grade mathematics (student safety), and improved ACT scores (total).

Among schools with higher concentrations of white students, we find only four significant impacts following wildfire closures: worse 4th grade mathematics and English, improved 7th grade mathematics, and improved 5th grade English scores (Supplementary Fig. 4). We again find significant impacts primarily among elementary school students following natural disaster closures: worse mathematics scores in 3rd, 4th, 5th, 6th, and 13th grades and worse English scores in 3rd, 4th, 6th, and 13th grades. Infrastructure closures are only associated with worse 6th grade English scores. Student safety closures are associated with worse 13th grade mathematics, 11th grade English, and SAT math, writing, and total scores. Total closures are associated with worse 4th grade English and mathematics, improved 7th grade mathematics, and improved ACT scores.

Overall, we find minimal to insignificant impacts of school closures from infrastructure, student safety, and total closures on exam scores at schools with higher concentrations of any subpopulation. By contrast, we find consistent significant impacts from wildfire and natural disaster closures, particularly on elementary school students.

#### Extended closures

To investigate the potential impacts of extended school closures, we rerun our analyses with dummy variables reflecting total days missed (1 day, 2 days, 3–5 days, 6–10 days, and 11 + days) from each closure cause. However, there are few cases of extended closures lasting more than 1 week (6 + days) from all causes except wildfires in our dataset, creating data limitations in our analysis.

Overall, we find significant impacts from short-term wildfire closures lasting under 1 week (1–5 days), primarily among younger students. We find declines in 3rd, 4th, 6th, and 13th grade mathematics following closures lasting between 1 and 10 days and declines in 4th grade English following closures lasting between 1 day and more than 2 weeks (11 + days). Closures lasting between 6 and 10 days are associated with improved SAT English, writing, and overall scores and with worse AP scores. We find minimal significant impacts from either short-term or extended closures across schools with higher concentrations of any subpopulation.

Schools with two weeks (6–10 days) of school closures from natural hazard impacts experience declines in 3rd, 4th, 11th, and 13th grade mathematics scores, while schools with only one closure day from natural hazard impacts experience improved English (3rd, 4th, 5th, and 13th grades) and mathematics scores (3rd, 4th, 5th, 6th, and 13th grades). We also find significant impacts of closures lasting between 6 and 10 days on schools with higher concentrations of certain subpopulations: worse 3rd and 13th grade mathematics (FRPMs); worse 8th grade English (Hispanic or Latino); worse 4th and 7th grade mathematics, worse 4th grade English, improved 5th grade English (African-American); improved 3rd grade mathematics, worse 8th grade English (Asian); and improved 8th grade English (white). We find no impacts of extended closures from natural hazard impacts on college preparatory exams.

Extended infrastructure closures (6–10 days) are associated with worse 4th grade mathematics, worse 7th grade English, and improved 11th grade English. Closures lasting between 6 and 10 days have significant impacts on schools with higher concentrations of specific subpopulations, primarily around middle school: worse 8th grade English (FRPMs); improved 8th grade English (Hispanic or Latino); worse 4th, 6th, and 13th grade mathematics, improved 8th grade English (African-American); improved 6th grade mathematics, worse 8th grade English (Asian), worse 6th grade mathematics, and improved 8th grade English (white). We find no impacts of closures lasting between 3 and 5 days from natural hazard impacts on college preparatory exams.

Student safety closures lasting between 6 and 10 days have significant impacts primarily on middle school students. Across all schools and among those with higher concentrations of low-income, Hispanic or Latino, and African-American students, school closures are associated with improved 8th grade English and mathematics scores. By contrast, among schools with higher concentrations of Asian and white students, extended student safety school closures are associated with worse 8th grade English and mathematics scores and improved 3rd grade English scores. We find no significant impacts on college preparatory exams across student safety closures or across schools with higher concentrations of any subpopulation for closures lasting 3–5 days.

Total closure days have significant impacts on scores primarily among younger students following extended closures, both overall and across subpopulations. Closures lasting between 6 and 10 days are associated with worse 4th, 6th, and 13th grade mathematics; worse 3rd and 4th grade English; improved SAT English, writing, and overall; and worse AP scores. We find fewer significant impacts when considering schools with higher concentrations of subpopulations. Following closures lasting 6–10 days, we find worse 7th grade mathematics (FRPMs); worse 5th and 7th grade mathematics, improved 3rd grade English, worse 5th grade English (Hispanic or Latino); improved 6th and 8th grade mathematics, improved 8th grade English, worse SAT mathematics (African-American); improved 7th grade mathematics, and worse 3rd grade English scores (white). Closures lasting more than 2 weeks (11 + days) are associated with few significant impacts, though these primarily affect 4th grade students: worse 4th grade English (overall); improved 4th and 13th grade English (FRPMs); improved 4th grade mathematics and English (Hispanic or Latino); and worse 4th grade mathematics and English (white). We find no significant impacts on college preparatory exams among specific subpopulations for closures lasting longer than 5 days.

#### Quarantine and outbreak closures

Prior research on the effects of school closures caused by disease outbreaks and quarantine procedures on student achievement is limited and, in the case of the COVID-19 pandemic, primarily relies on model projections. The dataset includes 72 cases of disease-related school closures (categorized under “student safety”) which lasted an average of 2.28 days (range: 1 to 5 days). Quarantine-related student safety closures are associated with worse 7th grade mathematics and English scores, but have no significant impact on college exam scores (Supplementary Fig. 6). We find five cases of improved scores among specific populations: 7th grade English (FRPMs), 3rd grade mathematics and English (Hispanic or Latino), 7th grade mathematics (African-American), and 13th grade English (white). We also find worse scores in 13th grade English among schools with higher concentrations of Hispanic or Latino students. Short-term quarantine closures lasting no more than 5 days generally have minimal negative impacts on academic achievement at schools with higher concentrations of any subpopulation.

#### Robustness of analysis

Multiple testing may accidentally produce false positive results by chance. With a more stringent alpha level of 0.00007 (instead of 0.05) using Bonferroni’s adjustment for multiple tests, we find insignificant results in 681 (98.7%) of 690 statistical tests (examining wildfire, natural hazard impacts, infrastructure, student safety, and total closures across all grades and college preparatory exams). We find significant results across only 9 statistical tests (wildfires: worse scores in 3th grade English (overall), worse scores in 13th grade mathematics (African-American); natural hazard impacts: improved scores in 4th and 7th grade English (African-American), improved scores in 5th grade mathematics (Asian), and worse scores in 13th grade mathematics (white); student safety: improved SAT mathematics scores (overall, FRPMs); and total: worse scores in 13th grade mathematics (overall)). However, the Bonferroni correction is conservative and may result in greater likelihood of failure to reject a false null hypothesis in cases of high numbers of tests, resulting in false negatives^37^.

## Discussion

School closures caused by wildfires and natural hazard impacts have significant impacts on academic achievement, primarily among elementary school students. By contrast, infrastructure and student safety closures have minimal or insignificant impacts on student performance across all ages, even when we specifically consider low-income or minority student populations or extended closures. These findings indicate that children may be quite resilient, at least in the short-term, to unanticipated school closures from infrastructure or student safety closures, or that teachers are able to adjust their lesson plans effectively following schoolwide closures. Our results extend prior research on the effects of schoolwide closures on academic performance, which has found divergent results ranging from insignificant to significant, negative impacts, particularly on low-income and minority students (Supplementary Table 1b)^24,25^.

However, extended closures caused by wildfires are associated with worse academic performance. Such extended closures are likely to become more common in California in future years, largely driven by more extreme wildfires during the fall semester^38–40^. We find that closures lasting more than one week from wildfires negatively affect scores, particularly among the youngest students in our dataset (3rd and 4th graders). However, data limitations prevent us from drawing strong conclusions on how extended school closures may affect academic performance. Future disasters could result in days to weeks of school closures, with a potentially compounding negative effect on student performance after repeated or extended school closures year after year. Future research could explore the impacts of cumulative school closures across multiple years on academic performance. Repeated, extended school closures in response to disasters would also likely have distinct impacts on students than the sustained, multi-month virtual schooling during the COVID-19 pandemic. Similarly, the effects of long-term school closures and virtual schooling from the COVID-19 pandemic will likely be more severe than our findings based on short-term closures from any cause, including quarantines.

Furthermore, the most significant effects of school closures may appear several years post-disruption^36,41^. Data limitations prevent us from generating strong conclusions about possible lagged effects from schoolwide closures beyond the immediate academic year. In addition, some school districts have petitioned to waive the CAASPP or have not participated in the exams following a wildfire in order to focus instead on missed curriculum. For example, some school districts in Santa Rosa did not take the CAASPP exams following the 2017 Sonoma Complex Fires, so our data are incomplete^42^.

Similarly, data limitations prevent us from examining the effects of schoolwide closures on academic performance at an individual student level. We did not have access to individual student academic performance data over time and therefore cannot make conclusive claims related to the impacts of schoolwide closures on individual students, which may contribute to our insignificant results. Future research may reveal differences between the impacts of schoolwide closures on aggregate schoolwide, grade-level academic performance compared to the impacts of schoolwide closures on a longitudinal study examining individual academic performance.

This study extends prior literature by examining both long-term events (such as the Camp Fire which closed 88 schools for more than 5 days) as well as minor events that cause short disruptions (such as a 1 day power outage or road closure). Most previous school closure studies have focused on either acute events like hurricanes and earthquakes^36^ or exclusively on common occurrences like snow days^24^. Our findings offer important parallels to COVID-19-related closures. Although we find minimal to insignificant impacts from quarantine closures, our data only include quarantine closures lasting up to 5 days compared to the months of virtual learning during the pandemic. Our null results related to quarantine closures compared to those from COVID-19 studies indicate that the pandemic is unique in its effects on learning and academic losses.

In addition, both COVID-19 and the four causes considered in this study can threaten students’ mental and physical health because students may experience secondary impacts such as disruptions to family economic security, housing stability, and trauma. Though we cannot isolate the educational impacts of closures from their broader societal impacts, there are important similarities between the pandemic and other school closure causes in how students may be responding to both learning disruptions and personal or community changes.

These findings also have important policy implications. California currently offers a waiver process for educational agencies to receive credit for missed school days and lost attendance resulting from an emergency, but schools are not required to make up missed school days^43^. The original language in the proposed S.B. 884 in the 2019–2020 California legislative session would have allocated funding to local educational agencies to make up instructional days lost to emergencies like wildfires in the event of five or more cancelled school days; that language was removed, and the bill ultimately failed^44^. Our findings that both short-term and extended wildfire closures are detrimental to student learning (especially among younger students) indicate that schools may need to make up classes even after brief closures, particularly elementary schools. Our results indicate that missed days from a particular closure cause may have a bigger impact on academic performance than total closure days.

In the event of an unanticipated closure, some schools have embraced remote learning to provide continuity outside of the classroom. For example, after the 2018 Camp Fire, schools in the Paradise Unified School District adjusted to an online learning platform^45^. During the COVID-19 pandemic, schools across the country transitioned to remote learning. Projections of the impacts of remote learning on students during the pandemic suggest high levels of learning loss and exacerbated levels of learning inequities, particularly among low-income, African-American, and Hispanic students, who are more likely to receive low-quality or no remote instruction^23,46,47^. Though our dataset does not include information on whether school closures resulted in virtual learning, research from the pandemic suggests that remote learning can result in lower levels of academic achievement among the most vulnerable student populations. Virtual learning may not be a solution for future school closures based on other research demonstrating the disproportionate negative impact of virtual learning on low-income and minority students, especially given the insignificant impacts of non-pandemic-related school closures on scores^23,46^.

Students may be resilient to school closures from non-disaster causes, or teachers can make up lessons effectively after schoolwide closures. However, the ability for children to recover from traumatic events like wildfires often depends on the emotional support from parents and caregivers like teachers or school counsellors^48^. As closures become more common, particularly from wildfires, schools should actively prepare for closures by supporting and training teachers to address student concerns and trauma within the classroom, while also recognizing the impacts of the closure cause on teachers themselves^5,49^. Teachers report struggling to respond to school demands after major local incidents^50^; more training on how to adjust curricula after school closures and on supporting students—especially elementary school students—could benefit both teachers and students after an unexpected school closure.

## Methods

### Data collection and cleaning

We received data from CalMatters’ Disaster Days dataset, which included school closure data between 2002 and 2019 in California as reported to the California Department of Education. Causes of school closure were pre-sorted by CalMatters in categories of wildfires, natural disaster & weather, student safety, infrastructure, and other. “Other” causes primarily included teacher strikes, faculty or administrative medical absences, days of mourning, and road closures. The dataset lists the school site, district name, county, school code, school year, type of disruption, and the details and dates of the disruption. According to the dataset, schools closed 15,112 times over 34,183 days in the seventeen year period between the 2002–2003 and 2018–2019 academic years^51^. We removed outliers of closures lasting more than 21 days to result in a total of 33,819 closure days. Based on the dates provided in the dataset, we identified closures as occurring in the fall or the spring semester, or, in some cases, both semesters (e.g., a disruption caused by the Thomas Fire resulted in school closures on December 7–15, 2017, and January 8–12, 2018). Closures in the fall semester were identified as occurring between August and December, and closures in the spring semester were identified as occurring between January and July.

We downloaded data on (1) Scholastic Assessment Test (SAT), (2) American Collegiate Testing (ACT), (3) Advanced Placement (AP), and (4) California Assessment of Student Performance and Progress (CAASPP) scores, as available from the California Department of Education. Average SAT scores by school were available in a consistent format between 2002–2003 and 2015–2016. The SAT test format changed after the 2003–2004 academic year from a 1600 point scale (a combination of verbal and mathematics) to a 2400 point scale (a combination of verbal, mathematics, and writing). We included scores from both the new and old point scales in our analysis^52^.

Second, average ACT scores by school were available between 2002–2003 and 2018–2019. ACT scores are a composite of average scores across reading, writing, mathematics, and science sections. The scores range from 1 to 36 points across each of the four sections, with the total score an average of the individual scores of the four sections^53^. We included average ACT scores in our analysis.

Third, AP scores by school were available between 2002–2003 and 2018–2019. AP exams are scored on a scale from 1 to 5. Scores of 1 and 2 are failing grades, while scores of 3, 4, and 5 are passing grades. Data included the percentage of exams falling into each scoring category from an individual school in addition to the percentage of passing exams (a composite of 3, 4, and 5 scores)^54^. We included percentage of passing exams in our analysis.

Finally, average CAASPP scores for both English Language Arts/Literacy and Mathematics were available for the academic years between 2014–2015 and 2018–2019. The CAASPP was established in 2014. Scores are available for students in grades 3, 4, 5, 6, 7, 8, and 11, in addition to all grades taking the test at an individual school, presented as grade 13 in the data. We present impacts on scores for grade 13 in our analysis, but they are inconclusive in regard to grade level scores and all grades taking the exam within a single elementary, middle, or high school. Overall scores for English Language Arts/Literacy and Mathematics are reported at “standard exceeded,” “standard met,” “standard nearly met,” and “standard not met.” For our analysis, we considered scores at the level of “standard not met.” Students also receive scores in four areas for English Language Arts (reading, writing, speaking and listening, and research/inquiry) and three areas for Mathematics (concepts and procedures, problem solving/modeling and data analysis, and communicating reasoning). Scores in these seven subareas are graded at “above standard,” “near standard,” and “below standard”^53^. In addition, though the CAASPP tests are required across California, some school districts sought waivers or elected not to take the CAASPP exams following a wildfire, so our data are incomplete. For example, schools in the Santa Rosa High and Santa Rosa Elementary School Districts missed between 10 and 15 days of school as a result of the 2017 fall Sonoma Complex Fires, but they did not take the CAASPP exams in the 2017–2018 academic year.

We also downloaded additional data for each available year on student demographics and socioeconomics associated with each school in California. These data included: male and female enrollment^34^; enrollment by race or ethnicity (options included Asian, Pacific Islander, Filipino, Hispanic or Latino, African American not Hispanic, White not Hispanic, American Indian or Alaska Native, Two or More Races, and Not Reported)^34^; English as a Second Language speakers^56^; rates of participation in free or reduced-price meal programs (FRPMs)^57^; and whether the school is a charter school or a magnet school^58^.

We reshaped and merged all datasets together to create rows of data for each individual school across multiple years. We labeled all schools in individual academic years without a school closure as having 0 closures for each cause. We compared missing data in all individual datasets with the merged dataset to address any mistakes and removed any duplicate or outlier rows. For example, we removed data in cases when the percentage of English as a Second Language speakers exceeded 100%.

### Statistical analysis

We began by investigating the relationship between school disruption and academic performance. Since we have a panel data of schools, we used two-ways linear fixed effects regression to control for school and year fixed effects. Therefore, we conducted within-school comparisons by contrasting how different numbers of school closure days affect each school’s academic performance. We recognize that the bulk of school closures included in the dataset were short-term closures (average: 2.44 days). We used the plm package in R to estimate our models. Specifically, we began with the following equation:

1 $$\gamma_{it} = \propto_{i} + \mu_{t} + \mathop \sum \limits_{k = 1}^{k} \beta_{k} X_{kit} + \varepsilon_{it}$$

where $\propto_{i} \;{\text{and}}\;\mu_{t}$ were school and time fixed effects. An individual school’s academic performance ($\gamma$) was a function of different causes of disruption experienced by schools and a set of school-level characteristics (*X*). We considered four types of disruption causes: wildfires, natural hazard impacts, infrastructure problems, student safety concerns, and other causes. We also separately considered total closure days from any cause. The school-level time-variant characteristics include percentage of school population who received free and reduced-price lunch; percentage of female enrollment; percentage of English as a Second Language (ESL) learners; percentage of Hispanic or Latino, Asian, and African-American enrollment; and total enrollment in school. Time-invariant covariates, such as whether a school was a magnet or charter school, were not in the equation as the characteristics would be captured by school fixed effects.

We extracted the coefficient for each disruption cause from each equation for scores. The dots (see Fig. 2) represent the size of the coefficient in each equation and the two vertical lines delineate the confidence intervals. The outer vertical lines (thinner and longer) show confidence interval two standard deviations from the mean. The inner lines (thicker and shorter) show confidence interval one standard deviation from the mean.

Next, we wanted to determine if the disruption cause had particularly impact on schools with more low-income students or a higher concentration of minority students given some prior literature identifying greater negative impacts from school closures on low-income and minority students (Supplementary Table 1b). For example, for wildfire disruptions, we modified the previous equation and added an interaction term:

2 $$\gamma_{it} = \propto_{i} + \mu_{t} + \mathop \sum \limits_{k = 1}^{k} \beta_{k} X_{kit} + \beta_{1} \left( {Wildfire\;disruption} \right) \times \left( {Percent\;free\;or\;reduced\;price\;meals} \right) + \varepsilon_{it} .$$

This equation specifically tests whether the impact of disruptions had a differential impact on schools depending on the socioeconomic makeup of their students. We used percentage of students receiving FRPMs as a proxy measure of a school’s socio-economic resources. Our expectation was that schools with more low-income students would experience greater negative impacts from wildfire disruptions than schools with fewer low-income students.

Similarly, we employed the above equation to test whether wildfire disruption had a differential impact on schools based on their racial or ethnic composition. We considered concentrations of Hispanic or Latino, African-American, Asian, and white students within schools.

3 $$\gamma_{it} = \propto_{i} + \mu_{t} + \mathop \sum \limits_{k = 1}^{k} \beta_{k} X_{kit} + \beta_{1} \left( {Wildfire\;disruption} \right) \times \left( {Percent\;Hispanic\;or\;Latino} \right) + \varepsilon_{it}$$

We measured our dependent variable (academic performance) by (1) the percentage who did not meet the standard for either English or Mathematics for CAASPP exams, (2) SAT and ACT scores, and (3) the percentage of students passing AP exams. For the CAASPP analysis, we computed separate equations for grade 3 to grade 11 students, as well as for all grades in the school (grade 13).

We then considered the possibility that the effects of a school closure are non-linear and may only appear in cases of extreme closures. We created five dummy variables to reflect total days missed (1 day, 2 days, 3–5 days, 6–10 days, and 11 + days). We repeated equations 1, 2, and 3 with the categorical variables.

Our dataset of student safety closures also includes a subcategory of “quarantine and outbreaks”. We separated out quarantine-related closures from non-quarantine-related student safety closures. We used equations 1, 2, and 3 as above to test the effects of having a school closure caused by illness or other student safety causes on academic achievement.

Finally, we performed Bonferroni’s adjustment for multiple tests. We used a stringent alpha level of 0.0007 because we ran 620 statistical tests (English and Mathematics scores and college preparatory exams for wildfires, natural disasters, infrastructure, student safety, and total closures days across overall closures and specific subpopulations).

## Limitations

Our study tracks individual schools over time rather than individual students. Therefore, we cannot make conclusive claims on the long-term or multiyear impacts of school closures on students as individuals, as students may move away from the school district or enter a new school for middle or high school. Instead, our results represent aggregate impacts on academic performance associated with specific school demographics (such as percentage of students receiving free or reduced-price meals) in conjunction with schoolwide closures in that particular year. In addition, as most schools have an even distribution between male and female enrollment, our reliance on aggregate data offers little indication of whether schoolwide closures influence male or female students differently. Similarly, the low percentages of students identifying as Pacific Islander, Filipino, American Indian or Alaska Native, and Two or More Races across schools were not high enough to generate statistically significant results and were not included in our analysis. Data issues prevented us from using percentage of dropouts in our statistical analysis, as many schools reported more than a 20% dropout rate (already twice the state average), and in many cases more than 100% of the students were reported as dropouts. This study only examines the impacts of school closures on public schools, including charter schools, and does not consider the possible impacts of school closures on private schools.

Data availability also presents a study limitation. The CAASPP replaced the Standardized Testing and Reporting (STAR) program in 2014, so CAASPP data were only available in the five academic years between 2014–2015 and 2018–2019. By contrast, data were available for ACT and AP scores for seventeen academic years and for SAT scores for fourteen academic years. We have fewer years of CAASPP scores available to analyze compared to the SAT, ACT, and AP scores.

School closures may occur throughout the year. Schools may administer the CAASPP no sooner than after two-thirds of the way through a school’s annual instructional days have been completed, and no earlier than the second Tuesday in January. CAASPP tests can be administered throughout the last day of instruction, but no later than July 15^59^. AP exams take place every year during two weeks in mid-May^60^. Students can take the ACT and SAT exams multiple times and throughout the year. For example, in 2020–2021, the SAT is offered in August, October, November, December, March, May, and June, and the ACT is offered in September, October, December, February, April, and June^61^. Our data reflect test scores which may have occurred throughout the academic year. In any given year, students may take these standardized tests prior to experiencing a school closure that academic year, particularly for the SAT and ACT exams which are offered in the fall semester in anticipation of college applications.

## Supplementary Information

Supplementary Information.

## Data Availability

Data are available at Miller, Rebecca; Hui, Iris (2021), “Annual School Closures and Standardized Test Scores in California 2003–2019”, Mendeley Data, V1, https://doi.org/10.17632/r89gjb658r.1.
