# Supplementary material for: Impact of short school closures (1–5 days) on overall academic performance of schools in California
Source: Sci Rep. 2022 Feb 8;12:2079. doi: 10.1038/s41598-022-06050-9 (PMC8826373; doi:10.1038/s41598-022-06050-9)
Supplement: Supplementary file 1 — Supplementary Information. [file 41598_2022_6050_MOESM1_ESM.docx]

**Supplementary Table 1.** Selected literature on educational impacts of unanticipated school closures caused by (a) disease outbreaks and (b) disasters, student safety concerns, and other causes.

(a)

| Authors | Paper Title | Disruption Type | Dependent Variable | Location | Study Type | Impact |
| --- | --- | --- | --- | --- | --- | --- |
| Bao et al. (2020) | Modeling Reading Ability Gain in Kindergarten Children during COVID-19 School Closures | COVID-19 Pandemic | Reading ability gain among kindergarten students | United States | Model | - Projected major decline in rate of reading ability gain |
| Cristakis, Cleve, and Zimmerman (2020) | Estimation of US Children’s Educational Attainment and Years of Life Lost Associated with Primary School Closures During the Coronavirus Disease 2019 Pandemic | COVID-19 Pandemic | Educational attainment and years of life lost from primary school closures | United States | Model | - Projected 13.8 million years of life lost (undiscounted estimate) |
| Bao et al. (2020) | Modeling Reading Ability Gain in Kindergarten Children during COVID-19 School Closures | COVID-19 Pandemic | Reading ability gain among kindergarten students | United States | Model | - Projected major decline in rate of reading ability gain |
| Haeck and Lefebvre (2020) | Pandemic School Closures May Increase Inequality in Test Scores | COVID-19 Pandemic | Math, reading, and science assessment scores by socioeconomic status quintile | Canada | Model | - Projected expanded score gap between highest and lowest SES quintiles |
| Kuhfeld et al. (2020) | Projecting the Potential Impact of COVID-19 School Closures on Academic Achievement | COVID-19 Pandemic | Expected learning gains in reading and mathematics | United States | Model | - Major reductions in learning gains for reading, worse reductions for mathematics |
| Azevedo et al. (2020) | Simulating the Potential Impacts of COVID-19 School Closures on Schooling and Learning Outcomes: A Set of Global Estimates | COVID-19 Pandemic | Expected learning loss | 157 countries | Model | - Loss of between 0.3 and 0.9 years of schooling and learning losses up to $10 trillion based on shutdown length |
| Meyers and Thomasson (2017) | Paralyzed by Panic: Measuring the Effect of School Closures during the 1916 Polio Pandemic on Educational Attainment | Polio Pandemic | Educational attainment | United States | Fixed effects | - Greater negative impacts among older children and in more affected areas |

**(b)**

| Authors | Paper Title | Disruption Type | Dependent Variable | Location | Study Type | Impact |
| --- | --- | --- | --- | --- | --- | --- |
| Groppo and Kraehnert (2016) | The impact of extreme weather events on education | Severe winters | Completed basic education | Mongolia | Difference-in-differences | - Impact unlikely |
| Goodman (2014) | Flaking Out: Student Absences and Snow Days as Disruptions of Instructional Time | Snow days | State assessment exams in mathematics and English language arts | Massachusetts, United States | Fixed effects and instrumental variables estimates | - Minimal to no impact |
| Marcotte and Hemelt (2008) | Unscheduled School Closings and Student Performance | Snow days | State mathematics and reading assessments | Maryland, United States | Panel data, fixed effects | - Worse negative impacts among younger students  - Largest reading declines at schools with lower SES |
| Andrabi, Daniels, and Das (2020) | Human Capital Accumulation and Disasters: Evidence from the Pakistan Earthquake of 2005 | Earthquake | Academic assessments of English, mathematics, and Urdu | Northern Pakistan | Fixed effects | - Limited impact on test scores |
| Thamtanajit (2020) | The impacts of natural disaster on student achievement: Evidence from severe floods in Thailand | Flood | School-level O-net examination scores | Thailand | Difference-in-differences | - Primarily negative, significant impact on scores for younger students  - Minimal significant impact on scores for older students |
| Morrill and Westall (2021) | Which students are most impacted by extended school closures? | Hurricane (Florence) | State mathematics and reading assessments | North Carolina | Difference-in-differences with school fixed effects | - Minimal impacts overall  - More significant impacts among older students  - More significant impacts among Black and lower SES students |
| Gibbs et al. (2019) | Delayed Disaster Impacts on Academic Performance of Primary School Children | Wildfire (2009 Black Saturday bushfires) | National primary school assessments | Australia | Multilevel models | - No significant impact for writing, spelling, and grammar  - Delayed reductions associated with reading and mathematics |
| Brück, Di Maio, and Miaari (2019) | Learning the Hard Way: The Effect of Violent Conflict on Student Academic Achievement | Exposure to violent events | Probability of passing final exam | West Bank | Fixed effects | - No significant impact |
| Belot (2010) | Do teacher strikes harm educational attainment of students? | Teacher strike | Educational attainment, class repetition | Belgium | Difference-in-Differences | - Greater negative impacts |
| Baker (2013) | Industrial actions in schools: Strikes and student achievement | Teacher strike | Elementary school curriculum-based standardized test scores | Ontario, Canada | Fixed effects | - Negative impacts among older elementary students  - Negative impacts on math scores |

**Supplementary Figure 1.** Effect of school closures caused by the interaction of closures and proportion of Hispanic or Latino students by closure cause on standardized test scores for (a) grades 3-11 and all grades (grade 13) for mathematics, (b) grades 3-11 and all grades (grade 13) for English, and (c) college standardized exams. Lines indicate confidence intervals two standard deviations from the mean. Note that the y-axis scale varies among plots.**
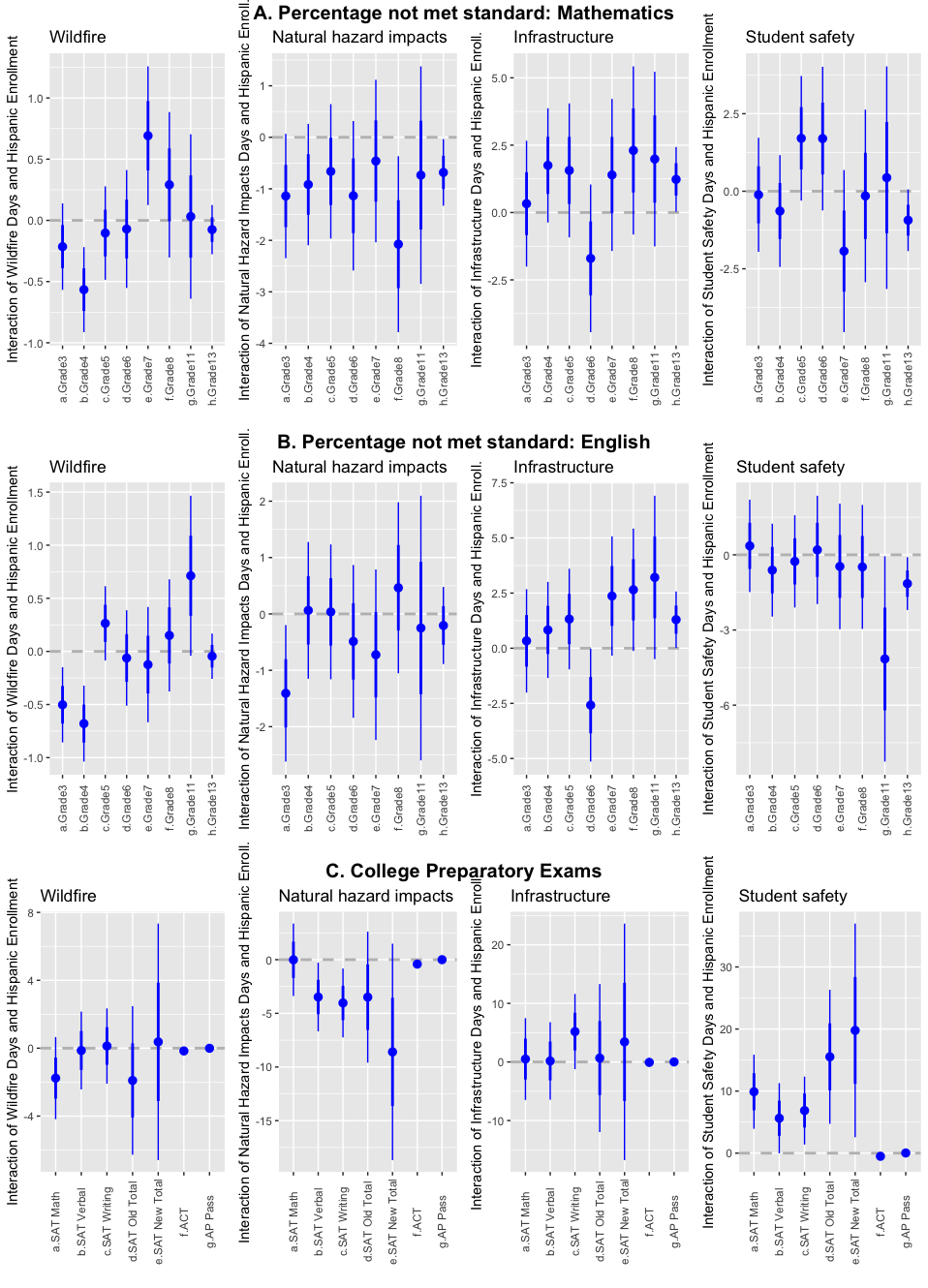
**

**Supplementary Figure 2.** Effect of school closures caused by the interaction of closures and proportion of African-American students by closure cause on standardized test scores for (a) grades 3-11 and all grades (grade 13) for mathematics, (b) grades 3-11 and all grades (grade 13) for English, and (c) college standardized exams. Lines indicate confidence intervals two standard deviations from the mean. Note that the y-axis scale varies among plots.

**
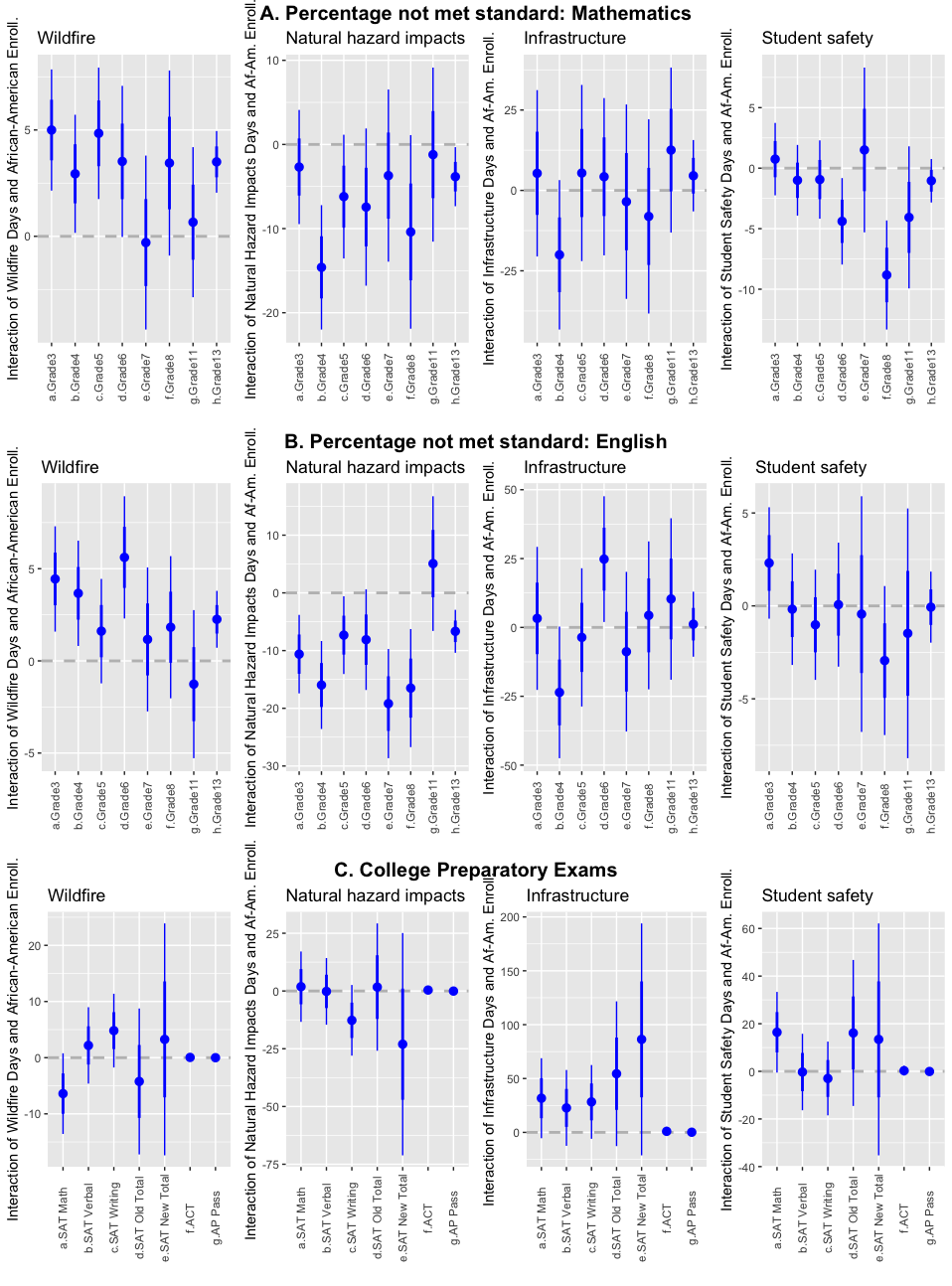
**

**Supplementary Figure 3.** Effect of school closures caused by the interaction of closures and proportion of Asian students by closure cause on standardized test scores for (a) grades 3-11 and all grades (grade 13) for mathematics, (b) grades 3-11 and all grades (grade 13) for English, and (c) college standardized exams. Lines indicate confidence intervals two standard deviations from the mean. Note that the y-axis scale varies among plots.


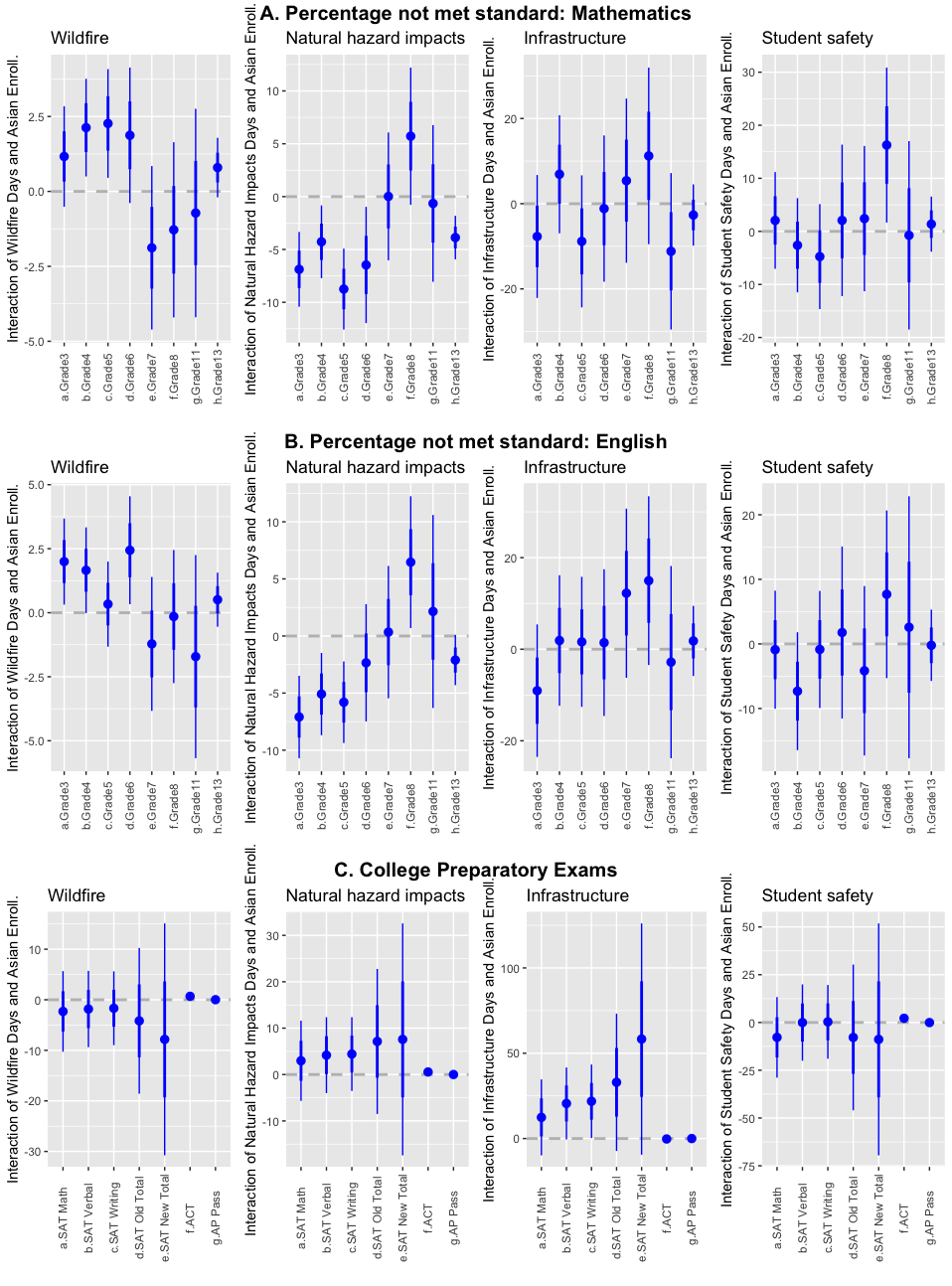


**Supplementary Figure 4.** Effect of school closures caused by the interaction of closures and proportion of white students by closure cause on standardized test scores for (a) grades 3-11 and all grades (grade 13) for mathematics, (b) grades 3-11 and all grades (grade 13) for English, and (c) college standardized exams. Lines indicate confidence intervals two standard deviations from the mean. Note that the y-axis scale varies among plots.

**
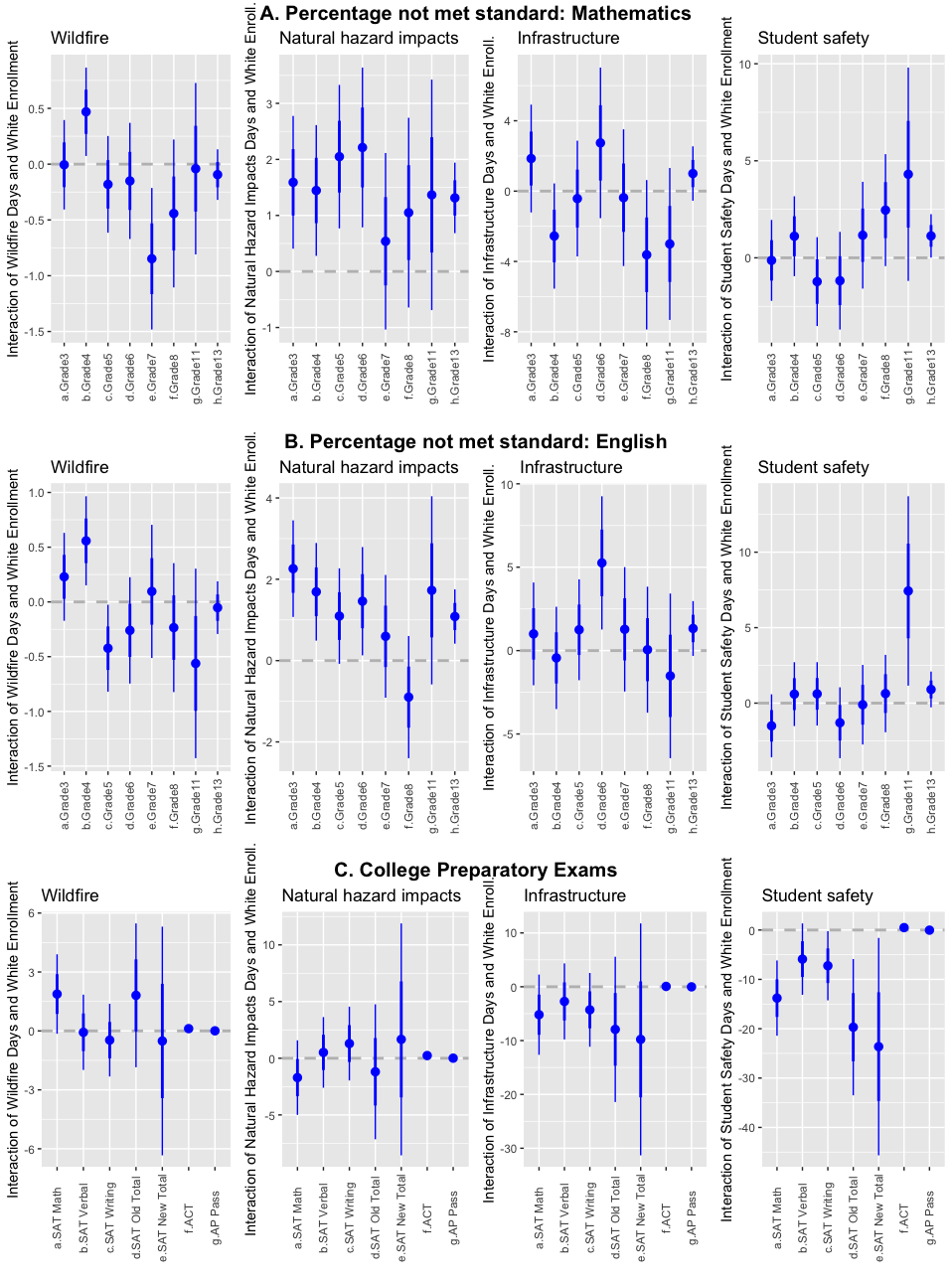
**

**Supplementary Figure 5.** Effect of school closures caused by total school closure days on standardized test scores and interactions of total closures and proportion of low-income, Hispanic or Latino, African-American, Asian, and white students for (a) grades 3-11 and all grades (grade 13) for mathematics, (b) grades 3-11 and all grades (grade 13) for English, and (c) college standardized exams. Lines indicate confidence intervals two standard deviations from the mean. Note that the y-axis scale varies among plots.

**
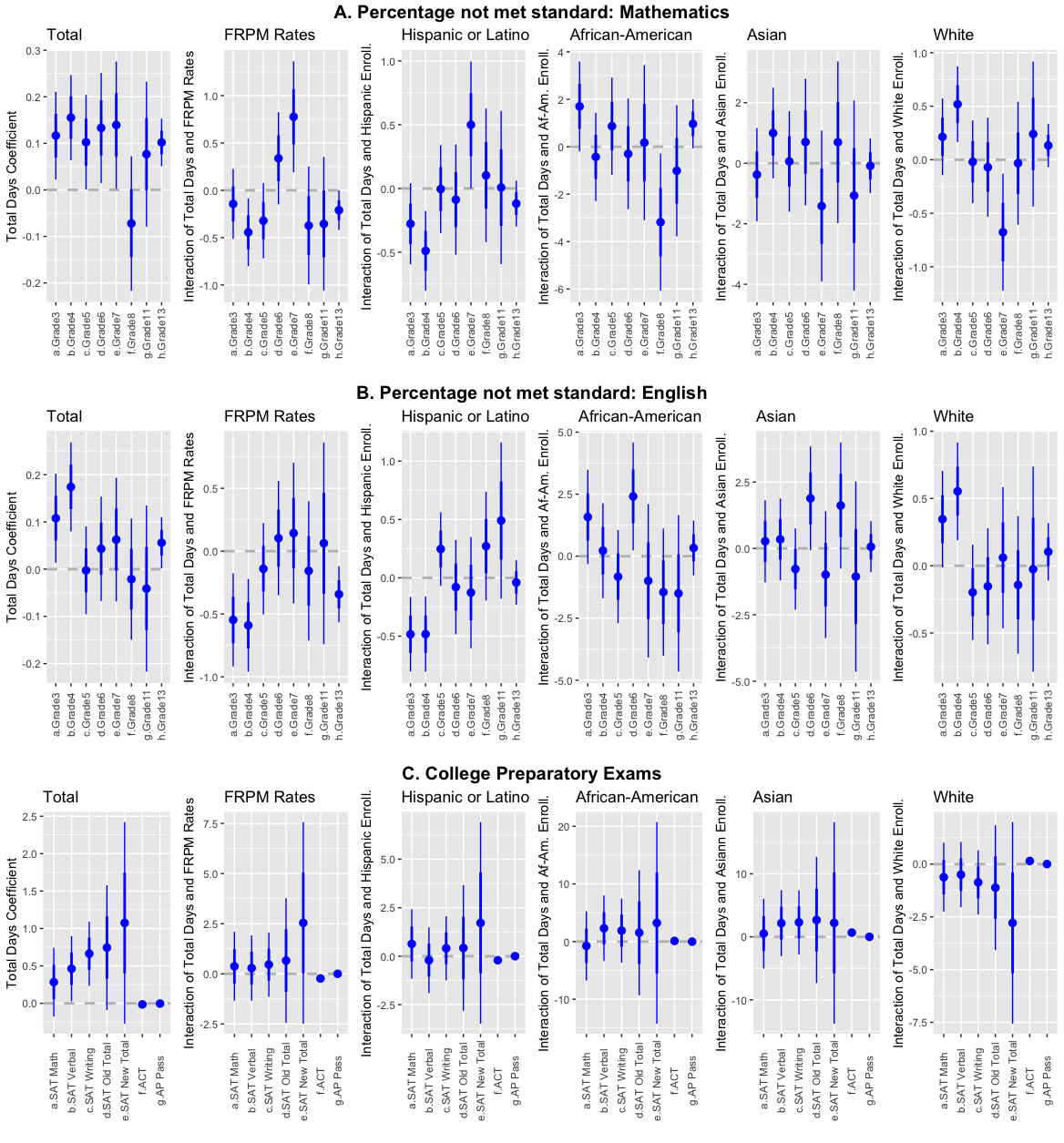
**

**Supplementary Figure 6.** Effect of school closures caused by the quarantine and outbreaks on standardized test scores and interactions of quarantine closures and proportion of low-income, Hispanic or Latino, African-American, Asian, and white students for (a) grades 3-11 and all grades (grade 13) for mathematics, (b) grades 3-11 and all grades (grade 13) for English, and (c) college standardized exams. Lines indicate confidence intervals two standard deviations from the mean. Note that the y-axis scale varies among plots.

**
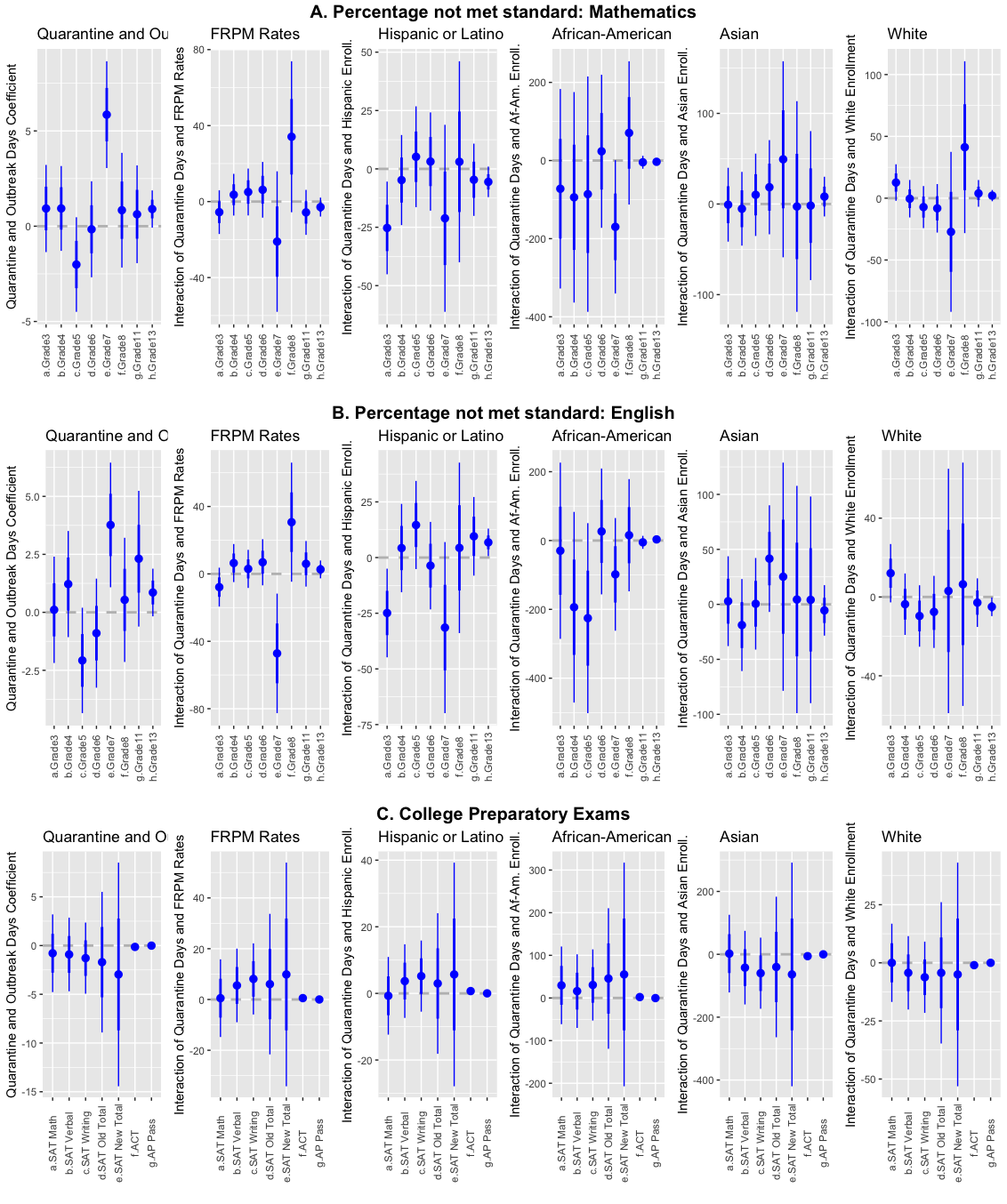
**
